# Sox2 Localization During Spermatogenesis and Its Association with other Spermatogenesis Markers Using Protein-Protein Network Analysis

**DOI:** 10.18502/jri.v24i3.13273

**Published:** 2023

**Authors:** Emad Reza, Hossein Azizi, Thomas Skutella

**Affiliations:** 1- Faculty of Biotechnology, Amol University of Special Modern Technologies, Amol, Iran; 2- Institute for Anatomy and Cell Biology, Medical Faculty, University of Heidelberg, Heidelberg, Germany

**Keywords:** Adult germline stem cells, Cell differentiation, Seminiferous tubules, Sox2 protein, Transcription factors

## Abstract

**Background:**

Sox2 (SRY box2) is an essential transcription factor that plays a vital role in spermatogenesis and regulates the genes in this process. Sox2 is important for pluripotency, self-renewal, and even spermatogonial stem cell differentiation. This gene is found in pluripotent and specialized cells, and it is involved in their biological activities.

**Methods:**

Protein-protein interaction (PPI) network analysis was performed during spermatogenesis using NCBI, STRING, and Cytoscape databases. Then, after isolating spermatogonial stem cells from 6 C57BL/6 mice, mouse embryonic stem cells and ES-like cells were prepared. In the following, Sox2 expression was examined in differentiated and undifferentiated spermatogonia by immunohistochemistry (IMH), immunocytochemistry (ICC), and Fluidigm PCR (polymerase chain reaction). Finally, the results were compared using the Kruskal-Wallis and Dunn tests at the significance level of p<0.05.

**Results:**

The results of this experiment showed that contrary to expectations, Sox2 has cytoplasmic expression in undifferentiated cells and nuclear expression in differentiated cells in *in vitro* conditions. In addition, the expression of Sox2 increased during differentiation. Fluidigm PCR showed a significantly higher expression of Sox2 (p<0.05) in differentiated compared to undifferentiated spermatogonia. Sox2 has an interaction with other genes during spermatogenesis such as Oct4, Nanog, Klf4, Stra8, Smad1, Tcf3, and Osm.

**Conclusion:**

Sox2, which is known as a pluripotency marker, has a vital role in spermatogenesis and could be a differential marker. Sox2 has strong connections with other genes such as Oct4, Nanog, Klf4, Tcf3, Osm, Stra8, Lim2, Smad1, Gdnf, and Kit.

## Introduction

Spermatogenesis is a complex process initiated by undifferentiated spermatogonia located on the basement membrane of the seminiferous tubules (1). During this process, regulated proliferation and differentiation of undifferentiated spermatogonia within the seminiferous tubules determine the long-term production of mature spermatozoa to transmit genetic information to the subsequent generation (2, 3). The undifferentiated spermatogonia themselves also undergo self-renewal continuously to maintain the progenitor state and population. A normal spermatogenesis process involves regulatory mechanics mainly controlled by endocrine signals from testicular somatic cells such as peritubular myoid cells (PTMs), Leydig cells, and Sertoli cells (SCs) (1). The spermatogonial stem cells (SSCs) express several stem cell markers, including Lin28, OCT4, Sall4, and SOX2 (4, 5), and their ability to self-renew implies that they are a form of pluripotent stem cells that might play a key role in organ regeneration (6). More than 2000 genes with a regulatory function for more than ten transcription factors are involved in pluripotency (7).

Sox2 is a transcription factor that plays an important role in maintaining pluripotency and differentiation of stem cells (8) and progenitor cells (9). In addition, it is also expressed in the inner cell mass (ICM) and ectoderm of blastocysts with a role in cell reprogramming (10). Sox2 can also convert somatic cells to pluripotent stem cells by regulating Sall4, Plzf, Gfra1, Oct4, Klf4, Foxm1, Cux1, Zfp143, Trp53, E2f4, Esrrb, Nfyb, and c-Myc (9, 11–13). These factors are involved in reprogramming and pluripotency and can produce induced pluripotent stem (iPS) cells by regulating each other (14, 15).

The expression of Sox2 and Nanog has also been detected in primordial germ cells of mice (16, 17). Sox2 in human primordial germ cells may still exist, although more investigation must be done for its detection. Table 1 shows a list of genes related with spermatogenesis; however, the association and co-expression of these genes with Sox2 is unknown. Previous studies have shown that when Sox2 expression is reduced, the number of pluripotent cells decreases and the cell differentiation starts (18). Sox2 has also been reported to show a high expression in some diseases, for example, brain cancers that can cause pituitary tumors (19), while a decreased Sox2 expression has been observed in patients with ocular abnormalities (20, 21). In addition, increased Sox2 expression has been correlated with breast cancer, colorectal cancer, and glioblastoma, while its decrease can lead to gastric cancer (22–25). In humans, the expression of Sox2 is different in various cell types and tissues. The lung, prostate gland, stomach, testis, and fallopian tube have high expression levels, while the bone marrow, endometrium, heart, kidney, liver, and pancreas show lower expression levels (26). The goal of this research was to assess the interaction of Sox2 gene with genes involved in spermatogenesis. In this study, gene expression was investigated and compared in spermatogonial stem cells in differentiated and undifferentiated types. Moreover, the quantity and mode of expression of this gene in differentiated and undifferentiated spermatogonia were studied. The ultimate objective of this research was to obtain a better understanding of the mechanisms involved in sperm generation and, as a result, to improve male infertility treatment.

**Table 1.** Some of the essential genes involved in the spermatogenesis process and their known functional roles in this process

**Gene**	**Full name and role**	**Ref.**
**Dnmt3b**	DNA methyltransferase 3b is essential for establishing DNA methylation patterns during the development and exacerbation of meiosis in spermatogenesis	(43)
**Irf3**	Interferon regulatory factor 3 is expressed during the transition from leptotene to pachytene spermatocytes	(44, 45)
**Egf**	Pro-epidermal growth factor plays a role during spermatogenesis in round spermatids	(46)
**Dnmt3a**	DNA methyltransferase 3a is required for methylation of most imprinted loci in germ cells and exacerbates meiosis in spermatogenesis	(47)
**Socs1**	Suppressor of cytokine signaling 1 plays a role in elongation of spermatid	(48–50)
**Lim2**	Lens fiber membrane intrinsic protein is present during spermatogenesis in round spermatids	(51)
**Gdnf**	Glial cell line-derived neurotrophic factor is expressed during spermatogenesis in round spermatids	(52)
**Efna2**	Ephrin-A2 plays a role in elongation of spermatid	(49)
**Egr2**	E3 SUMO-protein ligase may play a role in regulating hindbrain segmentation and transition from leptotene to pachytene spermatocytes	(53)
**Akap4**	A-kinase anchor protein 4 plays a role in sperm motility	(54)
**Stra8**	Stimulated by retinoic acid gene 8 protein is a meiosis inducer. That is required in male and female germ cells and plays a role in the exacerbation of meiosis in spermatogenesis	(55)
**Smad1**	Mothers against decapentaplegic homolog 1 expression in round spermatids may play a role in initiation and maintenance of spermatogenesis	(50, 56)
**Bmp8b**	Bone morphogenetic protein 8b generates primordial germ cells and plays a role in initiating and maintaining spermatogenesis	(57)
**Sim2**	Single-minded homolog 2 is expressed during development and transition from leptotene to pachytene spermatocytes	(50)
**Dad1**	Dolichyl-diphosphooligosaccharide-protein glycosyltransferase subunit 1 is expressed during spermatogenesis in round spermatids	(16)
**Rfx4**	This transcription factor plays a role in brain development and transition from leptotene to pachytene spermatocytes	(45)
**Kit**	Stem cell growth factor receptor plays a critical role in regulating cell survival, proliferation, and spermatogenesis	(53)
**Six2**	Homeobox protein Six2 is a transcription factor with an important role in the development of several organs and transition from leptotene to pachytene spermatocytes	(58)
**Mapk14**	Mitogen-activated protein kinase 14 is expressed during spermatogenesis in round spermatids	(59)
**Hoxa1**	Homeobox protein Hox-A1 is a transcription factor during the elongation of spermatid. It also plays an essential role in maintaining embryonic stem cells	(60)
**Fcgr2b**	Low-affinity immunoglobulin gamma Fc region receptor II is expressed during spermatogenesis in round spermatids	(50, 61)
**Tcf3**	Transcription factor E2-alpha is a transcriptional regulator and plays a role in transition from leptotene to pachytene spermatocytes	(45)
**Osm**	Oncostatin-M is a growth regulator and is expressed in transition from leptotene to pachytene spermatocytes	(62)

## Methods

### The PPI network establishment:

The genes involved in the spermatogenesis process were first identified in NCBI database and used in the STRING online databases v 11.5 to construct the PPI network. Then, the Cytoscape software v 3.9.1 (http://www.cytoscape.org/) was used to visualize and analyze the PPI network and screen necessary modules. The first neighbor’s nodes were identified with Sox2 as explained in the following sections.

### Gene Set Enrichment Analysis (GSEA):

The biological roles of the genes in the PPI network of neighbor’s nodes, identified by Sox2, were shown and functional enrichment analysis using STRING database by Cytoscape software, Gene Ontology (http://geneontology.org/), and Enrichr (GO_Biological_Process_2023, GO_Cellular_Component_2023, GO_Molecular_Function_2023, Reactome_2022, and KEGG_2019_Mouse) was performed. Several functional enrichments related to our data were also reviewed without considering a false discovery rate (FDR) value.

### Enzymatic digestion:

After being isolated from the animal, testicular cells from 6 C57BL/6 mice were put in a phosphate-buffered saline solution (PBS; Invitrogen, USA). Chopped tissue was placed in Hank’s balanced salt solution (PAA Laboratories, USA) containing Ca and Mg ions, as well as collagenase type IV enzyme (0.5 *mg/ml*), DNAse (0.5 *mg/ml*), and dispase (0.8 *mg/ml*). In fetal bovine serum or FBS, digestive enzymes were inhibited by 10%, and the resultant solution was gently pipetted to create a single-cell suspension. After washing and centrifuging the Nutrient Mixture F-12 (DMEM/F12) for 70 *min* at 1500 *rpm*, using a 70-micron nylon filter that allows Nutrient Mixture F-12 passage, the supernatant was removed and the residual cells were grown in a cell medium. Next, spermatogonia were grown in the laboratory (17).

### Culture of testicular cells:

Then SSCs that digested testicular cells were cultured in Germ Stem Cells (GSCs) culture media at 37°*C* with a 5% CO_2_ supplementation. The GSC culture media was composed of Stem-Pro-34 medium, 1% N2-supplement (Invitrogen, USA), 5 *μg/ml* of bovine serum albumin (Sigma Aldrich, USA), 1% L-glutamine (PAA laboratories, USA), 1% penicillin/streptomycin (PAA laboratories, USA), 20 *ng/ml* of epidermal growth factor (EGF) (Sigma Aldrich, USA), 30 *ng/ml* of estradiol (Sigma Aldrich, USA), 6 *mg/ml* of D-glucose (Sigma Aldrich, USA), 0.1% beta mercaptoethanol (Invitrogen, USA), 100 *μg/ml* of ascorbic acid (Sigma Aldrich, USA), 1% nonessential amino acids (PAA laboratories, USA), 30 *μg/ml* of pyruvic acid (Sigma Aldrich, USA), 60 *ng/ml* of progesterone (Sigma Aldrich, USA), 1% MEM vitamins (PAA laboratories, USA), 100 *U/ ml* of human LIF (MilliporeSigma, USA), 10 *ng/ ml* of FGF (Sigma Aldrich, USA), 8 *ng/ml* of GDNF (Sigma Aldrich, USA), 1% ES cell qualified FBS, and 1 *μl/ml* of DL-lactic acid (Sigma Aldrich, USA) (27, 28).

### Culture of ES-like and ES cells in mice:

MESC and ES-like cells were grown in ES media including KO-DMEM (or DMEM high glucose) based on solution volume, FBS 15%, MEM Non-Essential Amino Acid (MEM NEAA) solution 1%, L-glutamine 1%, Pen-Strep 1%, beta mercaptoethanol 0.1%, and LIF 1000 *g*/l. After 4 to 5 days of development, these cells had taken up a major portion of the flask. After washing with PBS and trypsin-EDTA for 3 *min*, MESC and ES-like cells were transferred to a fresh MEF feeder layer. Finally, 15% FBS was used to deactivate trypsin-EDTA.

### Immunohistochemical staining:

Male mice’s testicles were removed, washed in PBS, and preserved in 4% paraformaldehyde for roughly 24 *hr* at room temperature. Paraplast Plus was used to develop the dehydrated testis. A microtome was used to cut 10 *m* thick testicular tissue blocks. The portions were mounted on Superfrost Plus slides and stored at room temperature until they were needed. Before staining, all sections were paraffinized with xylene and hydrated in a decreased ethanol series. After heat-mediated antigen retrieval and incubation for 20 *min* at 95°*C* with sodium citrate buffer (pH=6) 10 *mM* or EDTA (pH=8) 1 *mM*, those not bound with 10% serum and 0.3% Triton X- in PBS were blocked and inactivated by 89.7%, and immunofluorescence labeling continued as stated above (27).

### Immunocytochemical staining:

For immunocytochemistry (ICC) staining, plates from 24 cells were utilized, and cells were grown. The cultivated cells were fixed with 4% paraformaldehyde, then washed with FBS and Tween 20. The cells were permeabilized with 0.1% Triton dissolved in 99.9% PBS before being treated for 24 *hr* with the primary antibodies (Sox2, ab 15830). This procedure was then repeated for goat anti-mouse IgG H&L (Alexa Fluor® 647; Abcam, UK) labeled secondary antibodies. The nucleus was stained for 3 *min* at room temperature with DAPI (4′, 6-diamidino-2-phenylindole) (0.2 *g/ml*), and the cells were then fixed with polyvinyl alcohol (Mowiol®; Sigma Aldrich, USA). The tagged cells were examined using fluorescence microscopy (Olympus BX51: Olympus Life Science, Japan) and confocal microscopy (Zeiss LSM 700; Carl Zeiss AG, Germany) with pictures captured using the Zeiss LSM-TPMT camera (28, 29).

### Fluidigm PCR analysis:

Fluidigm PCR (dynamic array chips) was done to measure the expression of the gene Sox2 (Mm00488369_s1) and the housekeeping gene GAPDH (glyceraldehyde-3-phosphate dehydrogenase) (Mm99999915_g1). GAPDH was used for the normalization of SSCs. Approximately, 100 cells from each sample were selected using a micromanipulator and lysed with a particular lysis buffer containing 9 *μl* RTPreAmp Master Mix (5.0 *μl* Cells Direct 2× Reaction Mix) (Invitrogen, USA), 0.2 *μl* Superscript III (Invitrogen, USA), 2.5 *μl* of 0.2× assay pool and 1.3 *μl* TE buffer, before freezing and storing at −80°*C*. Reverse transcription was performed at 50°*C* for 15 *min* using reverse transcriptase enzyme which was inactivated by heating at 95°*C* for 2 *min*. The cDNA was denatured at 95°*C* for 15 *s*. After that, the products were pre-amplified at 60°*C* for 4 *min* for 14 cycles. The pre-amplified products were diluted up to 5-fold and then analyzed by Universal PCR Master Mix and Taq-Man® Gene Expression Assays in a BioMark system. The targeted transcripts were quantified using TaqMan RT-PCR on the Biomark RT-quantitative PCR (qPCR) system (all samples were examined in two replicates). The Ct values achieved from the Biomark System were analyzed by Gen-Ex software and MultiD analysis (27, 29). In the current investigation, animal experiments were approved by the Ethics Committee of Amol University of Special Modern Technologies, Amol, Iran (Ir.ausmt.rec.1400.03).

### Statistical analysis:

Each of the previous experiments was carried out at least three times. In these experiments, the expression of Sox2 gene in 4 groups including MEF, differentiated and undifferentiated spermatogonia, and ES-like cells using SPSS *vs*. 26 (IBM, USA) was not normally distributed and therefore nonparametric tests such as Kruskal-Wallis and then Dunn test were used for further measurements. The difference between the groups was regarded statistically significant when p<0.05.

## Results

**Figure 1.**
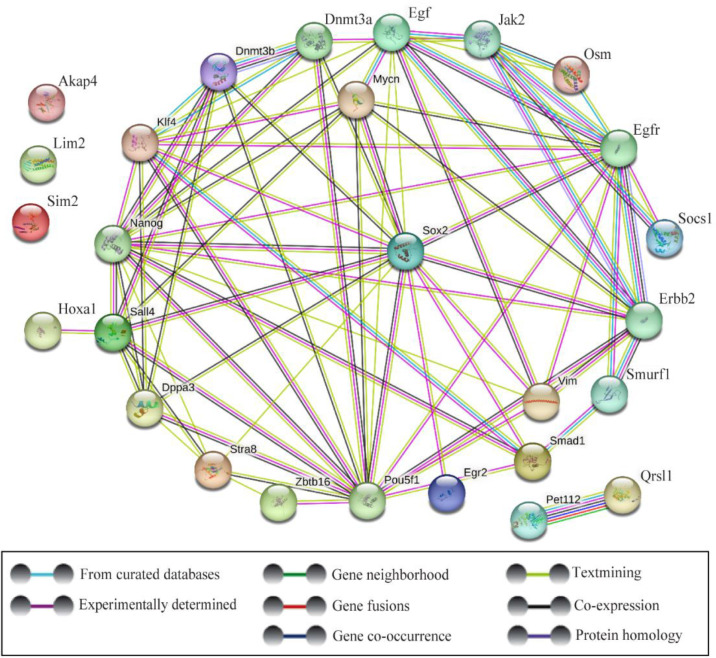
The protein-protein interaction network of Sox2 with other genes in the mouse using STRING database. The online database shows the network and connections of Sox2 during spermatogenesis

**Figure 2.**
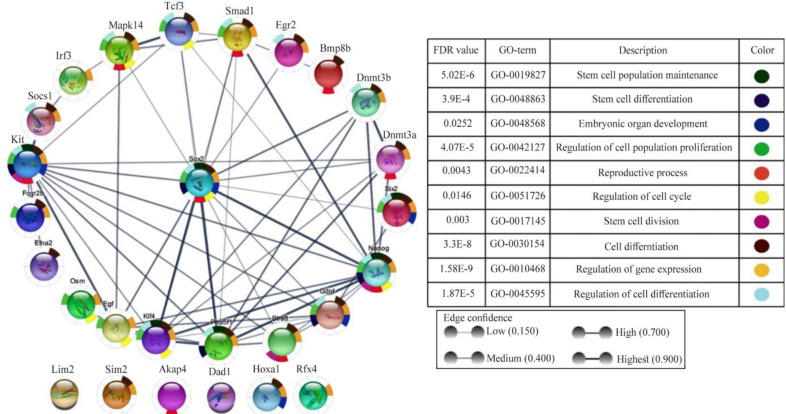
Networks of protein-protein interactions (PPIs) of Sox2 were constructed using the Cytoscape app. This app performed a simultaneous analysis of multiple protein networks with Sox2. This network shows the roles of proteins that interact with Sox2 in spermatogenesis and other activities in stem cell proliferation, development, and specialization. The network of Sox2 with other genes in spermatogenesis and their function are also shown

In this experiment, the network of Sox2 protein interaction and connection with some other proteins in spermatogenesis were analyzed as shown in figures 1 and 2. Key genes using STRING and Cytoscape databases were selected, and it was revealed that Sim2, Dad1, Hoxa1, Lim2, Akap4, and Rfx4 do not connect with Sox2 in regulation of expression. Figures 1 and 2 show the origins of these genes as well as the sources from which they were measured and connected, and where each of these genes is expressed in each testicle as well as their biological role. Furthermore, the genes that were more or less closely connected to one another were detected. Sox2 has a strong connection with Oct4, Nanog, and Klf4, but a poor connection with Mapk14, Smad1, Gdnf, Egr2, and Stra8, according to the results.

**Figure 3.**
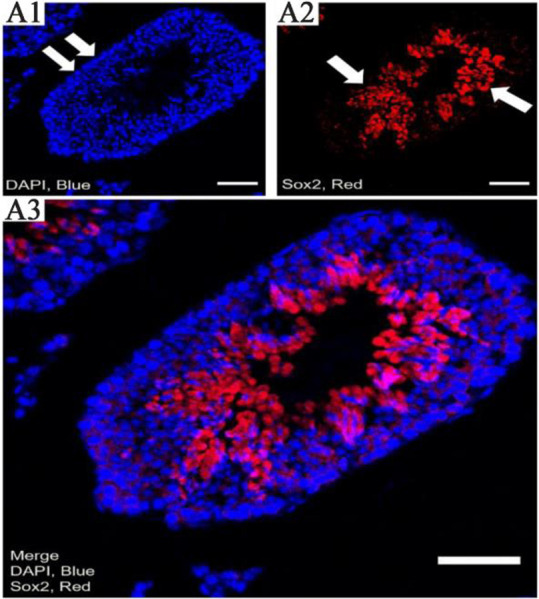
The pattern of Sox2 expression by confocal scanning UV-laser microscope. A1) the DAPI (blue for 4′, 6-diamidino-2-phenylindole) staining shows the nuclear cells, A2) the expression of Sox2 in seminiferous tubules, and A3) Merged images (scale bar 50 *μm*)

**Figure 4.**
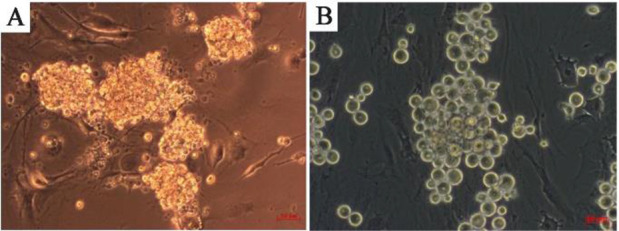
Differentiated (A) and undifferentiated (B) spermatogonia in bright field (scale bar 50 *μm*)

This is suggestive that Sox2 is connected with Pou5f1, Stra8, Klf4, Efna2, kit, Fogr2b, Socs1, Mapk14, Tcf3, Smad1, Egr2, Bmp8b, Dnm3b, Dnm3a, Nanog, and Gdnf in cell differentiation. In addition, Sox2 is connected with Pou5f1, Klf4, Kit, and Nanog in stem cell population maintenance. Sox2 expression was examined by semi-niferous tubules cross-section using immunohistochemical analysis. Immunohistochemistry analysis by confocal scanning UVlaser microscope demonstrated an increased nuclear expression of Sox2 in undifferentiated spermatogonia over time, during spermatogenesis *in vivo* (Figure 3). Single undifferentiated cells as well as a group of developed spermatogonial stem cells can be seen in this figure. The isolated spermatogonia, which were cultured and differentiated *in vitro*, presented different expressions of Sox2 (Figure 4). Considering Sox2 as a pluripotency factor, its expression in differentiated cells was increased beyond expectation.

**Figure 5.**
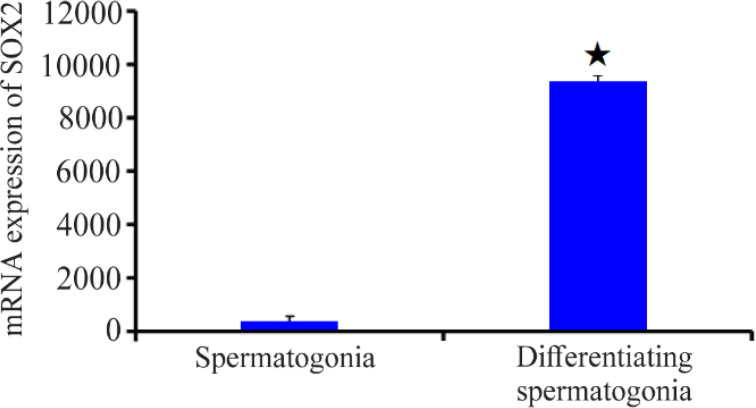
The mRNA expression of Sox2 in differentiated and undifferentiated spermatogonia. The Sox2 expression is significant (p<0.05) under *in vitro* conditions

mRNA analyses of spermatogonia cultivated *in vitro* revealed a much greater expression of Sox2 in differentiated spermatogonia than in undifferentiated spermatogonia according to Fluidigm PCR data (Figure 5). This finding demonstrates the high expression of Sox2 in differentiated cells as well as the significant difference in expression (p<0.05) between developed and undifferentiated cells.

**Figure 6.**
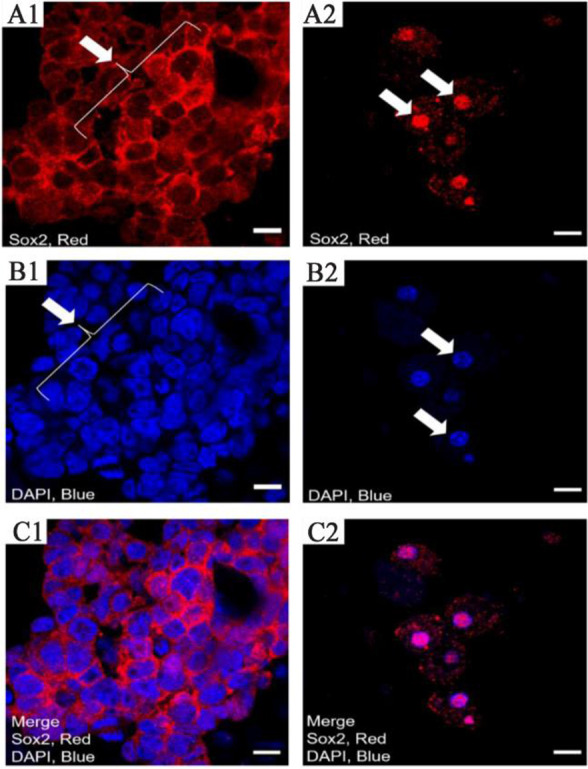
The pattern of Sox2 expression with immunocytochemistry in differentiated and undifferentiated cells of testis biopsy using confocal scanning UV-laser microscope. This Figure shows the expression of Sox2 in differentiated and undifferentiated cells under *in vitro* conditions. A) The expression of Sox2 in 1) undifferentiated and 2) differentiated spermatogonia. B1, 2) The DAPI staining for the nuclear cells and C1, 2) The merged images (scale bar 10 *μm*)

Moreover, high expression of Sox2 was observed in undifferentiated spermatogonia under *in vitro* conditions. On the other hand, Sox2 showed high nuclear expression in differentiated spermatogonia, similar to *in vivo* conditions (Figure 6). The study’s most interesting finding was that Sox2 exhibits high cytoplasmic expression in undifferentiated cells as well as high protein expression in differentiated cells.

## Discussion

It was found that Sox2 expression was essential to maintain pluripotency in differentiated spermatogonia. Campolo et al. (26) discovered that Sox2 is needed for primordial germ cell (PGC) development and pluripotency, and our findings reveal that differentiated spermatogonia have greater Sox2 expression than undifferentiated spermatogonia. The expression of this gene is significantly higher in differentiated cells than in un-differentiated cells, according to Fluidigm analyses. Furthermore, Campolo et al. reported that the expression of Sox2 affected spermatogenesis and increased during this process, which is in line with our findings of increased expression of Sox2 during the spermatogenesis process (26). The absence of Sox2 did not affect oogenesis and other types of oocytes (primary, secondary, and fully grown oocytes), despite its essential role in maternal to zygotic transition (26).

Sox2 has also been shown to be important in the promotion and development of the foregut endoderm, eye and inner ear neurogenesis (30, 31). The Cre/loxP recombination was used to investigate the conditional deletion of Sox2 in germ cells at different stages of development. The findings showed that embryos which lost Sox2 in PGCs had a significant decrease in the number of germ cells during differentiation. Regulation of gene expression is a critical factor in spermatogenesis process, and Sox2 deletion has been reported to affect PGC and spermatids (26, 32). Lack of Sox2 expression has also been shown to affect PGCs and resulted in fetal death (33). Sox2 is expressed during the developmental stage in PGCs, as predicted, according to immunohistochemistry data. This gene is required for germ cell proliferation, survival, and development. Sox2 is able to uncover chromatin, impact DNA, make it accessible for binding to other transcription factors, and even plays a role in sustaining transcription and self-renewal. Sox2 affects pluripotency and differentiation-induced gene expression, as well as phosphorylation, acetylation, ubiquitination, and methylation, according to a recent study (34, 35). Myers et al. (36) reported that mutant Sox2 can be replaced in mice, while Kim et al. (2021) (36) showed that mutant Sox2 with different mutations reduced the embryonic stem cell self-renewal potential. In addition, it has been found that Sox2 is essential for the growth in mice after fertilization (26). Also, other investigations demonstrated that Sox2 is expressed in amphibian oocytes (37).

The irregular expression of Sox2 in spermatogonial stem cells can lead to problems in the process of spermatogenesis and subsequent impaired fertility (26). *In vitro* and *in vivo* experiments revealed that Sox2 was expressed in SSCs, developed, and undifferentiated spermatogonia. Even though Sox2 is not expressed in human primordial germ cells, it is linked to regulation of genes involved in pluripotency, proliferation, and spermatogenesis process, including Kit, Tall, Rif1, and Zfp148 (PGCs) (38–40). Previous studies found that Sox2 had the best interaction with ATRX in neurons, where a role in chromatin remodeling and end transcription of genes and pluripotency was observed (41, 42).

In this study, genes presented in table 1 were used as keywords in STRING and Cytoscape databases. Some of the genes such as Hoxa1, Akap4, Rfx4, Lim2, Sim2, and Dad1 do not have a co-expression or correlation with Sox2 in the spermatogenesis process in mice, while each of them still has a separate vital role in this process. The goal of this research was to learn more about the mechanism of Sox2 expression in the testis during spermatogenesis and how other genes and transcriptional factors regulate it; moreover, the impact of Sox2 expression on maintaining and proliferating SSCs and spermatogenesis was assessed. The observed variations in *in vitro* and *in vivo* expressions of Sox2 during spermatogenesis are important results that will help researchers better understand how the process and features might impact male infertility and other disorders caused by Sox2 mutations. Further research on the regulation of Sox2 expression might lead to better treatments for male infertility and other transcription factors including ophthalmic defects and pituitary tumors.

## Conclusion

This study showed that Sox2 expression is essential for the pluripotency of stem cells and SSCs, while it also plays an essential role in the maintenance, increase, and specialization of SSCs and PGCs. In addition, spermatogonia obtained from *in vivo* and *in vitro* experiments showed distinct Sox2 expression. In *in vitro* experiments, Sox2 expression was greater in the nucleus of differentiated spermatogonia than in the cytoplasm of undifferentiated spermatogonia. In *in vivo* experiments, differentiated spermatogonia had considerably greater Sox2 expression than undifferentiated spermatogonia. Nonetheless, this gene expression was important for sustaining pluripotency in stem cells, which are necessary for spermatogenesis and play a critical role in the final phases. Further research regarding the role of Sox2 expression in spermatogenesis might be useful in improving male infertility treatments.

### Availability of data and materials:

The authors confirm that the data supporting the findings of this study are available within the article and its supplementary materials.
